# A Japanese Herbal Formula, Daikenchuto, Alleviates Experimental Colitis by Reshaping Microbial Profiles and Enhancing Group 3 Innate Lymphoid Cells

**DOI:** 10.3389/fimmu.2022.903459

**Published:** 2022-06-02

**Authors:** Zhengzheng Shi, Tadashi Takeuchi, Yumiko Nakanishi, Tamotsu Kato, Katharina Beck, Ritsu Nagata, Tomoko Kageyama, Ayumi Ito, Hiroshi Ohno, Naoko Satoh-Takayama

**Affiliations:** ^1^ Laboratory for Intestinal Ecosystem, RIKEN Center for Integrative Medical Sciences, Yokohama, Japan; ^2^ Laboratory for Immune Regulation, Graduate School of Medical and Pharmaceutical Sciences, Chiba University, Chiba, Japan; ^3^ Immunobiology Laboratory, Graduate School of Medical Life Science, Yokohama City University, Yokohama, Japan

**Keywords:** Japanese herbal medicine (Kampo medicine), Daikenchuto (DKT), biomolecular functions of herbal medicine, experimental colitis, Lactobacillaceae, gut microbiota, colonic homeostasis, group 3 innate lymphoid cells (ILC3s)

## Abstract

Daikenchuto (DKT) is one of the most widely used Japanese herbal formulae for various gastrointestinal disorders. It consists of *Zanthoxylum Fructus* (Japanese pepper), *Zingiberis Siccatum Rhizoma* (processed ginger), *Ginseng radix*, and maltose powder. However, the use of DKT in clinical settings is still controversial due to the limited molecular evidence and largely unknown therapeutic effects. Here, we investigated the anti-inflammatory actions of DKT in the dextran sodium sulfate (DSS)-induced colitis model in mice. We observed that DKT remarkably attenuated the severity of experimental colitis while maintaining the members of the symbiotic microbiota such as family Lactobacillaceae and increasing levels of propionate, an immunomodulatory microbial metabolite, in the colon. DKT also protected colonic epithelial integrity by upregulating the fucosyltransferase gene *Fut2* and the antimicrobial peptide gene *Reg3g*. More remarkably, DKT restored the reduced colonic group 3 innate lymphoid cells (ILC3s), mainly RORγt^high^-ILC3s, in DSS-induced colitis. We further demonstrated that ILC3-deficient mice showed increased mortality during experimental colitis, suggesting that ILC3s play a protective function on colonic inflammation. These findings demonstrate that DKT possesses anti-inflammatory activity, partly *via* ILC3 function, to maintain the colonic microenvironment. Our study also provides insights into the molecular basis of herbal medicine effects, promotes more profound mechanistic studies towards herbal formulae and contributes to future drug development.

## Introduction

Traditional Japanese herbal medicine, or Kampo medicine, originated in ancient China and was introduced to Japan and practiced since approximately the 6th century. Kampo medicine has evolved over the centuries and has been successfully integrated into the modernized medical system in Japan (1). In the 1980s, Japanese medical insurance approved 148 herbal preparations as prescribed medicines (1, 2). These preparations are mainly derived from several ancient Chinese medicine textbooks dated 2,000 years ago (1, 2). This management system guarantees the quality, purity, and safety of herbal medicines, providing preconditions for scientific research (1, 3). The management also makes herbal formulae more affordable and accessible to patients in need.

Herbal medicine also gained popularity in European countries and the U.S. three decades ago (4, 5). Approximately 20% of the U.S. population consumes herbal products (5). In addition, to date, herbal therapies still dominate primary health care in developing countries (6). However, the efficacy of herbal medicine is controversial due to the limited scientific evidence.

Daikenchuto (DKT) is one of the most frequently prescribed Kampo preparations for various digestive disorders (1, 3). It consists of *Zanthoxylum Fructus* (Japanese pepper), *Zingiberis Siccatum Rhizoma* (processed ginger), *Ginseng radix*, and maltose powder. Initially, DKT served as a prokinetic agent to improve gut motility in several clinical settings. A number of clinical trials have suggested that DKT enhanced the intestinal transit in patients who underwent abdominal surgeries and mechanical ventilation (7–9). In addition, clinical observations have indicated that oral intake of DKT for several months prevents the need for reoperation in patients with a subtype of inflammatory bowel disease (IBD) (10). Later reports have proposed that the herbal formula’s prokinetic actions may be due to its anti-inflammatory actions (11). More recently, several animal studies have revealed that DKT exerts anti-inflammatory activities by suppressing Akt and NF-κB pathways and interleukin (IL)-6 and enhancing adrenomedullin in gut epithelial cells (12–14). DKT has also been studied in the dextran sulfate sodium (DSS)-induced colitis models. Matsunaga *et al.* found that DKT resulted in higher serum hemoglobin concentrations and IL-10 levels compared with DSS-treated mice (15). DKT also reduced visceral pain and eosinophilic infiltration into the colon in a rat DSS colitis model (16). U.S. researchers have also developed considerable interest in DKT (17, 18). Additionally, a multi-center clinical trial of DKT has been performed, aiming for the FDA approval (ClinicalTrials.gov Identifier: NCT01607307).

IBD, which encompasses Crohn’s disease and ulcerative colitis, is a chronic gut inflammatory disorder thought to be caused by inappropriate immune responses. It is usually a life-long condition that can be treated with several medications, although it is difficult to achieve a complete cure. IBD has become a global health concern as we have seen a large surge in its prevalence in North America and northern Europe. IBD morbidity is also on the rise in newly industrialized regions such as Asia, the Middle East, South America, and Africa. The number of individuals with IBD worldwide almost doubled in the last twenty years, increasing from 3.7 million to 6.8 million by 2017 (19, 20). Drugs for IBD include 5-aminosalicylic acid (5-ASA) compounds, antibiotics, systemic corticosteroids, immunosuppressors, monoclonal antibodies, and inhibitors of tumor necrosis factor. Although these drugs have tremendously improved the outcome of IBD treatment, individual responses vary, and adverse events and the high cost of several of these medications are still problematic (20, 21). Hence, new therapeutic options balancing cost-effectiveness and potent anti-inflammatory effects for IBD are desired (20, 21).

Intestinal tissue-resident innate lymphoid cells (ILCs) are key mediators in gut homeostasis and might serve as novel therapeutic targets in IBD management (22). For instance, group 1 ILCs (ILC1s) contribute to the pathogenesis of IBD in humans and mice by producing interferon-γ (22). Group 3 ILCs (ILC3s) are potent immune cells for pathogen clearance in the intestine by secreting cytokines IL-22 and IL-17, although their role in IBD is still elusive (23, 24).

Previous observations in human and animal disease models imply that DKT resolves local inflammation in the gut by several mechanisms (10, 14). Thus, it leads us to speculate that DKT can serve as another anti-inflammatory agent complementary to the standard IBD therapies. Nevertheless, the studies regarding DKT were inconclusive due to the lack of molecular evidence. In particular, in-depth immunological insights into DKT and its therapeutic effects on colitis are largely undetermined yet.

Here, we investigated the impacts of DKT on the DSS-induced colitis model, with particular emphasis on its effects on gut microbiota and immune cell profiles. We demonstrated that DKT accelerated the recovery from experimental colitis in mice, accompanied by improvements in several colitis-associated features of gut microbiota, their metabolites, and the colonic ILC3 population. Our study highlights previously unrecognized effects of DKT on the immune-microbiota axis in colitis, and our work opens a future therapeutic opportunity for IBD patients.

## Results

### DKT Contributes to a Swift Recovery of DSS-Induced Colitis

**Figure 1:**
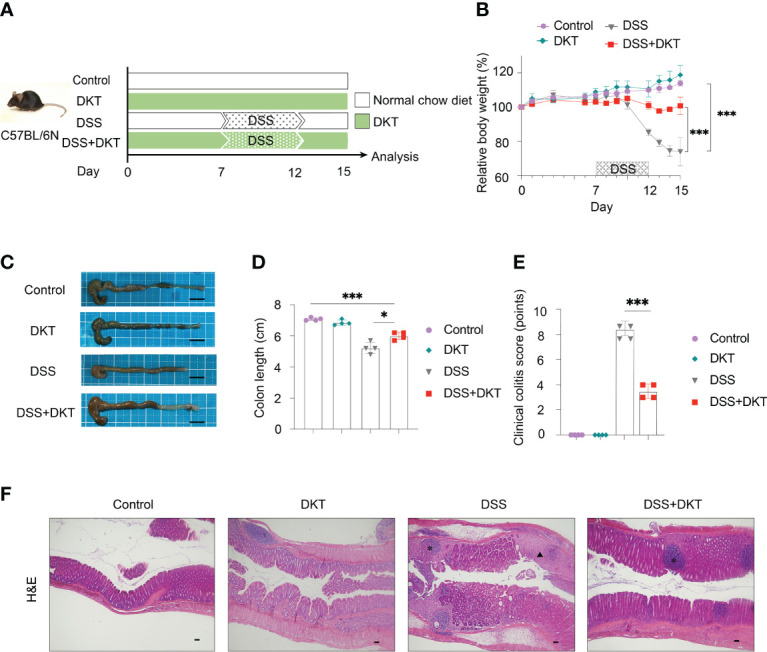
DKT alleviates the symptoms and pathology of dextran sodium sulfate (DSS)-induced acute colitis. **(A)** Experimental schematic: C57BL/6 mice were divided into four groups: a normal chow diet group (Control), a group given 5% DKT extract mixed in normal chow diet (DKT), a 2.5% DSS-induced colitis group (DSS), and a group of 2.5% DSS-colitis mice fed the same diet as the DKT group (DSS+DKT). 2.5% DSS in drinking water was administered beginning on day 7 and continued for 5 days, followed by a three-day DSS-free period. The mice were sacrificed on day 15. **(B)** Body weight changes over the course of the experiment (N=4 per group). **(C)** Representative macroscopic images of the colon post-mortem (N=4 per group). Scale bars represent 1 cm. **(D)** Colon lengths were measured from the proximal colon to the rectum post-mortem (N=4 per group). **(E)** Clinical colitis scores of the four experimental groups on day 15, measured in a blind fashion (N=4 per group). **(F)** Representative histology of H&E-stained longitudinal sections of the rectal colon (Control N=4, DKT N=4, DSS N=5, DSS+DKT N=5). Scale bars represent 100 μm. *indicates the isolated lymphoid follicles. The triangle indicates the ulceration. Each symbol **(D, E)** represents data from an individual mouse. Results are representative of two or three independent experiments with three to four mice in each experimental group. Graph **(B, D, E)** show means ± SEM; *p<0.05, and ***p<0.001. Statistical analysis was performed using One-way ANOVA with Tukey’s multiple comparisons test.

We first evaluated the efficacy of DKT on DSS-induced colitis. To this end, we set the following four experimental groups: a normal chow diet group (Control), a group given 5% DKT extracts mixed in a normal chow diet (DKT), a 2.5% DSS-induced colitis group (DSS), and a group of 2.5% DSS colitis mice that treated with 5% DKT (DSS+DKT) (**Figure 1A**). The body weight of the DSS group dropped sharply after two days of the DSS challenge until the day of sacrifice (day 15), whereas the DSS+DKT group almost maintained the starting body weight (**Figure 1B**). As expected, the body weight of the Control and DKT groups increased slightly throughout the experiments. The DSS+DKT group displayed a significantly longer colon than the DSS-treated mice (**Figures 1C, D**). Consistently, the DSS+DKT group showed significantly lower clinical colitis scores (**Figure 1E**). Histological analysis indicated that the DSS+DKT group had less epithelial damage than the DSS group, although hyperplasia was slightly presented in this group (**Figure 1F**). By contrast, the DSS group showed severe mucosa damage, such as multiple ulcerations, hyperplasia, edema, and inflammatory cell infiltration into the submucosa region. No apparent changes in the colon were observed in the Control and DKT groups. Overall, these results suggest that DKT treatment significantly prevents colonic damage in DSS-induced colitis; these observations prompted us to further interrogate the underlying molecular basis for the anti-inflammatory activities of DKT in colitis.

### DKT Ameliorates Colonic Dysbiosis by Increasing Lactobacillaceae and Propionate

**Figure 2:**
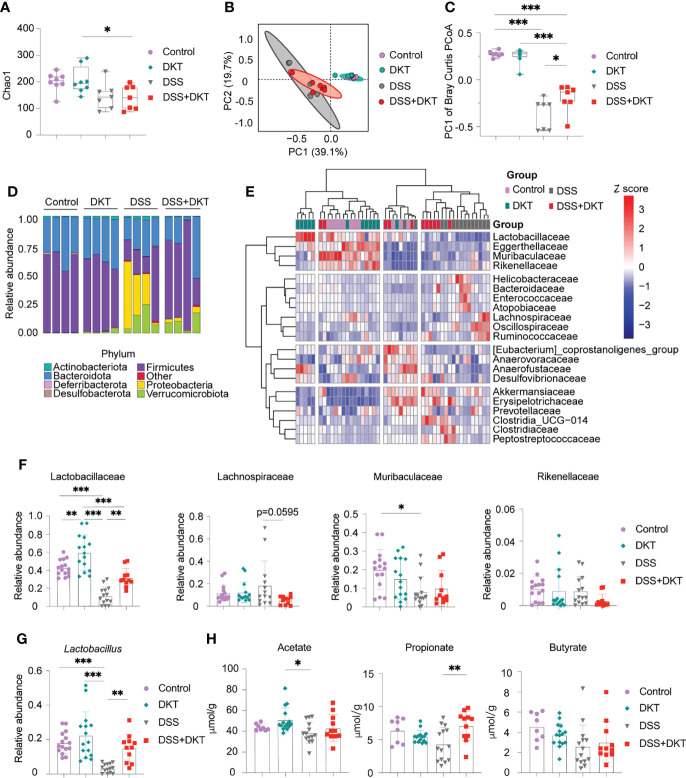
DKT alters the colonic microbiota composition and metabolites in DSS-induced acute colitis. **(A)** Chao1 alpha diversity of the colonic microbiota on day 15 (Control N=8, DKT N=8, DSS N=7, DSS+DKT N=7). **(B)** Principal coordinates analysis (PCoA) of the Bray Curtis distances between samples; day 15 samples are shown (Control N=8, DKT N=8, DSS N=7, DSS+DKT N=7). The ellipses indicate 95% confidence levels for the four groups. **(C)** PC1 of the Bray-Curtis PCoA among the four groups (Control N=8, DKT N=8, DSS N=7, DSS+DKT N=7). **(D)** The bacteria composition in the colonic contents at the phylum level on day 15 (N=4 per group). **(E)** Heatmap showing the top 21 highly abundant family-level bacteria on day 15 among the four groups. The microbial composition data at the family level were scaled and clustered based on the Ward-linkage method (Control N=11, DKT N=12, DSS N=14, DSS+DKT N=11). **(F)** Relative abundance of the family-level bacteria Lactobacillaceae, Lachnospiraceae, Muribaculaceae and Rikenellaceae on day 15 are shown (Control N=15, DKT N=15, DSS N=14, DSS+DKT N=11). **(G)** Relative abundance of the genus-level bacteria *Lactobacillus* on day 15 among the four groups (Control N=15, DKT N=15, DSS N=14, DSS+DKT N=11). **(H)** The concentration of three short-chain fatty acids (SCFAs), acetate, propionate and butyrate, on day 15 in the colonic contents are shown (Control N=8, DKT N=15, DSS N=14, DSS+DKT N=11). The SCFAs were measured by a GCMS platform. Results are pooled from two or three independent experiments with three to five mice in each experimental group. Each symbol **(A–C **and **F–H)** represents data from an individual mouse. Graphs **(A, C**, and **F–H)** display means ± SEM; *p<0.05, **p<0.01, and ***p<0.001. Statistical analysis was performed using One-way ANOVA with Tukey’s multiple comparisons test.

Previous evidence suggested that gut microbiota plays a pivotal role in DSS-induced colitis as it affects the sensitivity of DSS-induced colitis (25). In addition, short-chain fatty acids (SCFAs), the most abundant gut microbial metabolites, are deeply involved in the pathogenesis of colitis (26). We, therefore, asked whether DKT could impact the gut microbiota and their metabolites to alleviate the DSS-induced colitis. We first analyzed the gut microbiota composition by 16S rRNA amplicon sequencing. Both DSS and DSS+DKT groups showed lower taxonomic diversity represented by Chao1 index after colitis induction (**Figure 2A**), suggesting that DKT did not restore the loss of bacterial diversity that accompanies DSS colitis. However, the bacterial landscape of the four groups displayed unique features, as shown in the principal coordinates analysis of Bray-Curtis dissimilarity (**Figure 2B**). Dietary DKT itself did not affect the bacterial compositions compared to the Control group, while the DSS challenge shifted the colonic bacterial community apart from these two groups negatively along PC1. Although DSS and DSS+DKT groups were not clearly separated, the DSS+DKT group was closer to the non-colitis groups along PC1 (**Figure 2C**). In line with this result, taxonomic microbiota profiles at the phylum level showed that DKT restored the abundance of Firmicutes to levels similar to the non-DSS groups. In addition, Proteobacteria, the well-known pathobionts that facilitate colitis (27), were enriched in the DSS group while they were substantially reduced in the DSS+DKT group (**Figure 2D**). We next used the unsupervised hierarchical clustering based on Ward’s method to further characterize the similarities of the microbiota composition at the family-level among the four experimental groups (**Figure 2E**). Although the groups were not perfectly separated, the DSS group was distantly located from the non-DSS groups; the DSS+DKT group was largely located between the DSS and non-DSS samples. These findings imply that DSS administration vigorously altered the gut bacterial community and DKT blunted such alterations.

In the heatmap (**Figure 2E**), the top cluster of family-level bacteria, including Lactobacillaceae, Eggerthellaceae, Muribaculaceae, and Rikenellaceae, were the most prominently reduced by DSS administration. We further found that Lactobacillaceae, which belongs to the phylum Firmicutes and exerts various beneficial effects on the host physiology, including attenuation of gut inflammation (28), showed substantial alterations among the four experimental groups (**Figure 2F**, left panel). DSS challenge suppressed Lactobacillaceae while DKT administration partially restored their abundance. Consistently, DKT administration restored the abundance of *Lactobacillus*, a major genus-level bacteria of Lactobacillaceae, to levels similar to the non-DSS groups (**Figure 2G**). Of note, dietary DKT increased Lactobacillaceae compared to the control group (**Figure 2F**, left panel). In addition to the effects on Lactobacillaceae, the DSS+DKT group displayed a tendency of decreased Lachnospiraceae, which also belongs to the Firmicutes phylum and is the third most abundant family in the non-DSS groups, compared with the DSS group (**Figure 2F**). Lachnospiraceae plays a controversial role in human physiology and is known to contribute to the onset of inflammation and several metabolic disorders (29, 30). We did not observe any apparent alterations in other families (**Figure 2F** and **Supplementary Figure 1**).

We next performed a fecal metabolome analysis with particular emphasis on SCFAs. Notably, propionate, a major SCFA derived from the colonic bacteria, was significantly higher in the DSS+DKT group than the DSS group, and its concentration was comparable to the two healthy groups (**Figure 2H**). DKT did not affect acetate or butyrate. Collectively, these data reveal that DKT restores several critical features of DSS-induced colitis in terms of gut microbiota and their metabolites.

### DKT Preserves the Integrity of the Colonic Epithelial Barrier by Restoring Goblet Cells and Promoting Antimicrobial Peptides

**Figure 3:**
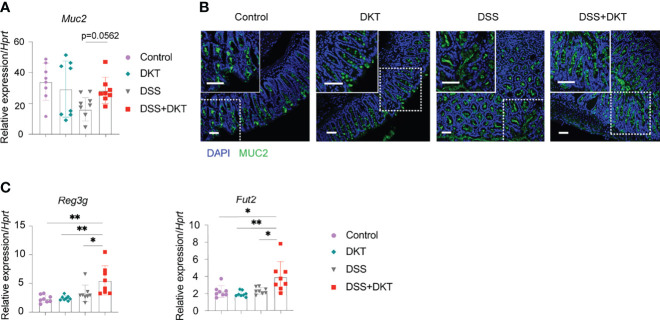
DKT protects the integrity of colonic epithelium by upregulating *Muc2*, the antimicrobial peptide gene *Reg3* and the fucosyltransferase gene *Fut2*. **(A)** *Muc2* mRNA expression in the colonic enterocytes after colitis induction (N=8 per group). **(B)** Immunofluorescence images of MUC2 expression (green) and DAPI (blue) staining in the rectal colonic tissue (Control N=3, DKT N=3, DSS N=4, DSS+DKT N=4). Scale bars represent 50 μm, inset scale bars represent 50 μm. **(C)** *Reg3g* and *Fut2* mRNA expression in the colonic enterocytes after colitis induction (N=8 per group). Each symbol **(A, C)** represents data from an individual mouse. **(A, C)**, results are pooled from two independent experiments with four mice in each experimental group and show means ± SEM; *p<0.05, **p<0.01. Statistical analysis was performed using One-way ANOVA with Tukey’s multiple comparisons test.

Once colitis strikes the community of microorganisms, it impairs mucus properties, and the microorganisms may reach the epithelium and affect its barrier function (31). MUC2 mucin, secreted by goblet cells, is a critical component of the gastrointestinal mucus layer to protect the epithelium (32). Previous studies also suggested that *Lactobacillus* could upregulate the MUC2 mucin (33, 34). Since we found that DKT maintained *Lactobacillus* under the DSS challenge, we asked how the MUC2 mucin and goblet cells were affected. We found that *Muc2* expression in colonic epithelial cells tended to be higher in the DSS+DKT group than in the DSS group (**Figure 3A**). Immunohistochemistry analysis of MUC2 showed no apparent differences between the two healthy groups, while the DSS+DKT group appeared to have restored MUC2 compared to the DSS group (**Figure 3B**). Goblet cells in the DSS+DKT group also appeared to increase due to the hyperplasia of the colonic glands, whereas DSS-treated mice showed significantly depleted and disrupted goblet cells (**Figure 3B**).

In addition to the mucus layer, antimicrobial peptides and fucosylation are other critical regulators in maintaining intestinal homeostasis and are deeply involved in the pathogenesis of colitis (35, 36). In this regard, we observed that a representative antimicrobial peptide gene *Reg3g* and an essential epithelial fucosylation gene *Fut2* mRNAs were significantly upregulated in the DSS+DKT group compared with the DSS group (**Figure 3C**).

Altogether, these findings reveal that DKT improves epithelial integrity in DSS-induced colitis, which may explain the ameliorated colitis symptoms in the DSS+DKT group.

### DKT Enhances ILC3 to Promote the Host Defense Against Experimental Colitis

**Figure 4:**
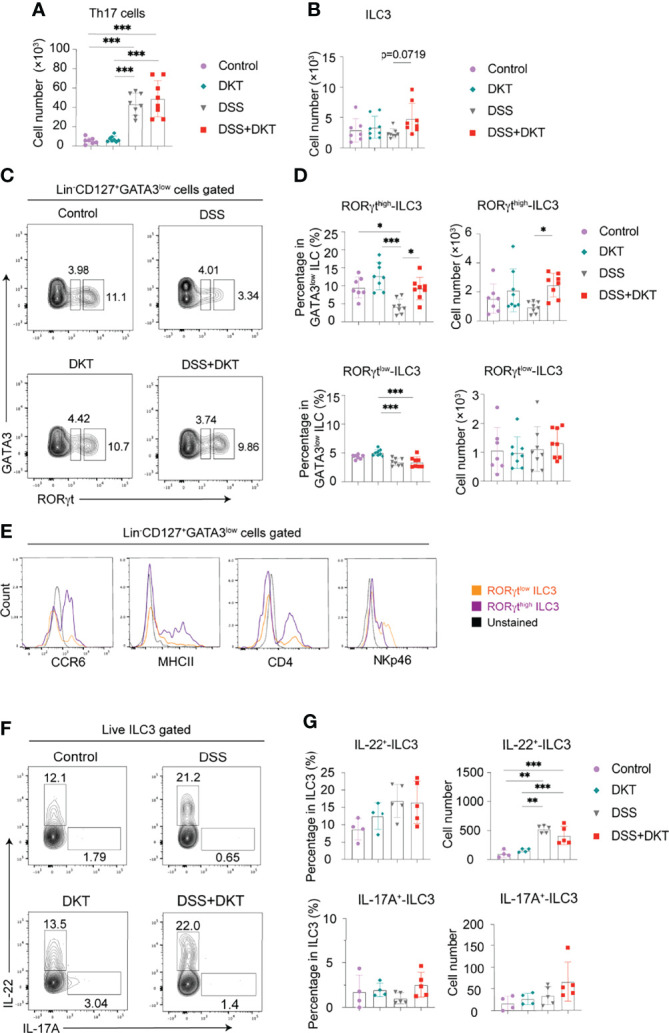
DKT significantly increases colonic RORγt^high^-ILC3 which resembles CCR6^+^ ILC3. **(A)** Absolute cell numbers of colonic Th17 cells isolated from the lamina propria region (Control N=7, DKT N=8, DSS N=8, DKT+DSS N=8). **(B)** Absolute cell numbers of colonic ILC3s isolated from the lamina propria region (Control N=7, DKT N=8, DSS N=8, DKT+DSS N=8). **(C)** Representative flow cytometry plots for gating colonic ILC3, and the two subpopulations of RORγt^low^ and RORγt^high^-ILC3s based on the intensity of the transcription factor RORγt. See **Supplementary Figure 2A** for details on the gating strategy. **(D)** Frequency and absolute cell numbers of RORγt^high^ and RORγt^low^-ILC3s (Control N=7, DKT N=8, DSS N=8, DKT+DSS N=8). **(E)** Representative histogram plots showing the expression of the surface markers CCR6, MHCII, CD4, and NKp46 by naive colonic ILC3s to discriminate the two ILC3 subpopulations, NKp46^+^ ILC3 (or NCR^+^ ILC3) and CCR6^+^ ILC3 (or LTi-like cell) (N=6). **(F)** Representative flow cytometry plots for IL-22^+^- and IL-17A^+^-producing ILC3s of the four experimental groups using *Rorc^GFP/+^* mice. **(G)** Frequency and absolute cell numbers of IL-22^+^- and IL-17A^+^-producing ILC3s (Control N=4, DKT N=4, DSS N=5, DKT+DSS N=5). Each symbol **(A, B, D, G)** represents data from an individual mouse. **(A–D)**, results are pooled from two independent experiments with three to four mice in each experimental group. Graphs **(A, B, D, G)** show means SEM; *p<0.05, **p<0.01, and ***p<0.001. Statistical analysis was performed using One-way ANOVA with Tukey’s multiple comparisons test.

It has been previously reported that secretion of antimicrobial peptides and fucosylation on epithelial cells are regulated by IL-22 (37, 38), which is secreted by several types of lymphocytes from the adaptive and innate immune systems, such as effector CD4^+^ T lymphocytes, particularly Th17 cells, and ILC3s (39, 40). The observed upregulation of *Reg3g* and *Fut2* by DKT in the colonic epithelial cells (**Figure 3C**) prompted us to interrogate the involvement of IL-22 in DKT-mediated amelioration of colitis. We first examined Th17 cell and ILC3 populations by flow cytometry. Colitis significantly increased the Th17 numbers and there was no difference between the DSS and DSS+DKT groups (**Figure 4A**). By contrast, the population of tissue-resident ILC3s tended to increase in the DSS+DKT group compared to the DSS group (**Figure 4B**). We further noted that DSS treatment suppressed the ROR-related orphan receptor gamma t (RORγt) intensity, as its expression level was lower than the other three experimental groups (**Figure 4C**). DSS decreased the RORγt^high^ expressing ILC3s, while DKT treatment significantly restored the frequency and cell numbers of RORγt^high^-ILC3s to levels similar to the non-DSS groups (**Figures 4C, D**: upper panel). There were no differences in RORγt^low^-ILC3s between the DSS and non-DSS groups (**Figure 4D**: lower panel). Moreover, DKT did not impact other subtypes of ILCs, such as ILC1s/natural killer (NK) cells or ILC2s (**Supplementary Figure 2B**). Although DSS-induced colitis led to the expansion of CD4^+^ T cells, B cells, and macrophages, DKT did not influence these populations (**Supplementary Figure 2C**). And conventional dendritic cells (DCs) remained unchanged among the four groups (**Supplementary Figure 2C**).

ILC3s have been reported to be quite heterogeneous and to encompass at least two major subsets. One subgroup expresses the surface marker NKp46, termed natural cytotoxicity receptor (NCR)^+^ ILC3s (40). The other subset expresses the chemokine receptor CCR6, as well as CD4 and MHCII, and these cells are termed lymphoid tissue inducer (LTi)-like cells (41, 42). Of note, differences in RORγt expression levels have been previously suggested to correlate with the different subtypes of ILC3s in the mouse small intestine (43). In order to further characterize the RORγt^high^-ILC3s in the mouse colon, which was significantly different between the DSS and DSS+DKT groups (**Figures 4C, D**), we next assessed the expression of surface markers such as CCR6, MHCII, NKp46, and CD4 to discriminate the ILC3 subgroups among naïve cells. RORγt^high^-ILC3s predominantly expressed CCR6, MHCII, and CD4, while RORγt^low^-ILC3s showed a higher level of NKp46 expression (**Figure 4E**). Taken together, these observations indicate that a large proportion of RORγt^high^-ILC3s primarily consists of the LTi-like phenotype, while the RORγt^low^-ILC3s mainly comprise NCR^+^ ILC3s.

We next assessed IL-22 and IL-17A-producing colonic ILC3s in *Rorc^GFP/+^* mice (**Figure 4F**). In steady-state, ILC3s produced a certain level of IL-22, although they barely produced IL-17A (**Figure 4G**). Despite the increase in IL-22^+^-ILC3s under the colitis condition, the cell number remained comparable between the DSS and DSS+DKT groups. IL-17A^+^-ILC3s did not differ among the four experimental groups. Similarly, *Il22* and *Il17a* mRNA levels in RORγt^high^-ILC3s in *Rorc^GFP/+^* mice were unchanged in all four groups (**Supplementary Figure 2D**).

SCFAs act as ligands for G-protein coupled receptors (GPCRs), including GPR41, GPR43, and GPR109A, to activate signaling cascades that exert anti-inflammatory activities in IBD (44). We, therefore, qPCR-quantified the expression of mRNAs encoding these GPCRs in the sorted RORγt^high^-ILC3s. We detected GPR43-encoding *Ffar2* mRNA in RORγt^high^-ILC3s in steady-state, and DSS treatment upregulated *Ffar2* expression, although there was no difference between the DSS and DSS+DKT groups (**Supplementary Figure 2E**). By contrast, the expression of GPR41-encoding *Ffar3* and GPR109a-encoding *Hcar2* was barely detected (comparing the scale of Y-axes in **Supplementary Figure 2E**), and the expression of *Ffar3* and *Hcar2* was unchanged regardless of DKT or DSS treatment (**Supplementary Figure 2E**).

Taken together, these data provide evidence that DKT impacts the colonic immune system, particularly by increasing the RORγt^high^-ILC3 subpopulation in DSS-induced colitis. We also show that this RORγt^high^-ILC3 population phenotypically resembles LTi-like cells, although their effector functions remain to be investigated.

### ILC3 Is a Critical Protective Immunoregulator in Experimental Colitis

**Figure 5:**
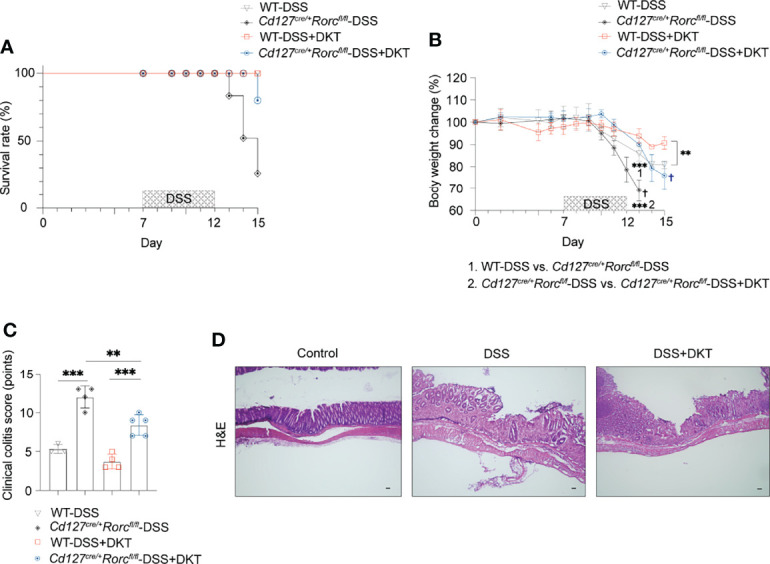
ILC3 knockout (KO) leads to a high fatality rate from DSS-induced colitis that could be partially blunted by DKT. **(A)** *Cd127^cre/+^Rorc^fl/fl^* mice, or ILC3 KO mice and their corresponding *Cd127^+/+^Rorc^fl/fl^*, or wildtype (WT) littermates underwent DSS treatment as described in **Figure 1A**. The survival rate for the four experimental groups is shown (WT-DSS N=3, *Cd127^cre/+^Rorc^fl/fl^*-DSS N=4, WT-DSS+DKT N=4, *Cd127^cre/+^Rorc^fl/fl^*-DSS+DKT N=5). **(B)** Body weight changes of the four experimental groups (WT-DSS N=3, *Cd127^cre/+^Rorc^fl/fl^*-DSS N=4, WT-DSS+DKT N=4, *Cd127^cre/+^Rorc^fl/fl^*-DSS+DKT N=5). “1” refers to the significantly lower body weight in the ILC3 KO mice compared to their WT littermates after DSS treatment on day 13. “2” indicates that the body weight of the DSS-treated ILC3 KO mice was significantly lower relative to the ILC3 KO mice treated with DSS+DKT on day 13. † indicates death of the mice. **(C)** Clinical colitis scoring of the four experimental groups on day 13 (WT-DSS N=3, *Cd127^cre/+^Rorc^fl/fl^*-DSS N=4, WT-DSS+DKT N=4, *Cd127^cre/+^Rorc^fl/fl^*-DSS+DKT N=5). **(D)** Representative light micrographs of colon sections of the ILC3 KO mice on day 15 that stained with H&E (Control N=2, DSS N=1, DSS+DKT N=4). Scale bars, 100 μm. Each symbol in graph **(C)** represents data from an individual mouse. Graphs **(B, C)** show means ± SEM; **p<0.01, and ***p<0.001. Statistical analysis was performed using One-way ANOVA with Tukey’s multiple comparisons test.

The above-described increase in RORγt^high^-ILC3s prompted us to test if these cells are involved in DKT-mediated alleviation of DSS-induced colitis. We took advantage of ILC3-specific knockout (KO) mice, generated by crossing CD127-Cre and *Rorc^fl/fl^* mice. While ILC3-sufficient wildtype (WT) mice survived DSS treatment regardless of DKT, ILC3 ablation resulted in a mortality rate of 83.3% in DSS-induced colitis. On the contrary, DKT treatment strikingly reduced the mortality to 16.7% (**Figure 5A**). Since under the experimental colitis condition, ILC3 KO mice started to die on day 13, we analyzed the body weight on day 13 instead of day 15 in this experimental setting. DSS-treated ILC3 KO mice showed a significantly lower body weight than their WT littermates (**Figure 5B**). The beneficial effect of DKT on DSS-induced body weight loss was, albeit partially, cancelled in the absence of ILC3, since the body weight loss of ILC3 KO mice upon DKT treatment was not as severe as that without DKT treatment (**Figure 5B**). Consistently, DKT treatment partially improved the clinical colitis score in ILC3 KO mice, although those mice suffered from more severe colitis symptoms than their WT littermates (**Figures 5C, D**). Collectively, these observations suggest that ILC3s play a critical role in attenuating DSS colitis and that DKT could ameliorate the severity of colitis at least partly through ILC3s.

## Discussion

The current study revealed novel biomedical functions of DKT extracts. We show that DKT significantly blunts DSS-induced acute experimental colitis by altering gut bacterial composition, increasing SCFAs, and sustaining colonic RORγt^high^-ILC3s, which could collaboratively maintain epithelial integrity (illustrated in **Supplementary Figure 3**). To the best of our knowledge, this is the most comprehensive study to characterize the molecular basis of the anti-inflammatory actions of DKT. The role of ILC3s in IBD has remained controversial, as they have been shown to exhibit both protective and pathogenic effects in the development of colitis (45). Our study thus provides a rationale for further clinical studies of DKT as a promising therapeutic option for IBD patients.

We found that DKT increased Lactobacillaceae and its genus-level bacteria *Lactobacillus*, which is beneficial to human health as it exhibits antimicrobial actions and promotes nutrient acquisition (46). *Lactobacillus* has also been shown to be an immunoregulator to resolve experimental colitis and can inhibit several pathogens *in vitro* (47, 48). Furthermore, DKT enhanced the microbial-derived SCFA propionate in the colon. This is consistent with previous studies showing that *Lactobacillus*, together with several other bacteria, contributes to intestinal fermentation and increases gut metabolites, including acetate, propionate, and lactate (49, 50). Together, these findings imply that the effects of DKT on experimental colitis are partly attributable to the promotion of colonic microbe symbiosis and enhancement of beneficial metabolites.

Our experiments further uncovered fundamental immune functions that DKT exerts to ameliorate experimental colitis. We conducted an extensive examination of the immune cell profiles in the colon, including both innate and adaptive immune cells. We found that only the RORγt^high^-ILC3 subset was significantly higher in the DSS+DKT group than the DSS group. These findings revealed that DKT ameliorates experimental colitis partially by modulating the ILC3 subset. The importance of ILC3s in colitis was corroborated by the experiment using ILC3 KO mice, where DSS induced a higher mortality in the KO mice compared to WT littermates. Meanwhile, DKT significantly reduced the mortality rate of ILC3 KO mice treated with DSS. This result also indicated that DKT ameliorates DSS colitis partly in an ILC3-independent manner. However, the underlying mechanisms are unknown and await further studies.

ILC3s are highly abundant in the gastrointestinal tract and promote metabolic and immune homeostasis by sensing and conveying cues from the luminal microbial community to the lamina propria (41). Nevertheless, their functions in colitis have not been well characterized (51). In this regard, our results show that colonic ILC3 plays an essential role in regulating the immune responses during colitis, supporting the hypothesis that ILC3 may serve as a potential therapeutic target to overcome intestinal inflammations (51). However, our data did not show significant differences in IL-22 or IL-17A production by ILC3s in DKT-treated mice. This is possibly due to our experimental scheme of evaluating the ILC3 phenotypes in the recovery phase rather than in the acute inflammatory phase and the transiently altered cytokine productions cannot be captured (52).

ILC3s are a heterogeneous population whose biological functions are highly tissue-specific (41). Our data demonstrate that DSS challenge suppressed the RORγt expression in ILC3s, whereas DKT administration reversed this phenomenon. Furthermore, this RORγt^high^-ILC3 subpopulation shares similarities with CCR6^+^ ILC3s, the predominant ILC3 subset in the colon (41). Based on our observations and a previous study (43), we extrapolate that the different RORγt expression levels may represent different ILC3 subpopulations, as RORγt^high^-ILC3s may consist of a large proportion of CCR6^+^ ILC3s and RORγt^low^-ILC3s contain mainly NCR^+^ ILC3s. This finding may further link the RORγt expression level to the unique effector functions of the two ILC3 subsets (53). The characteristics of RORγt^high^-ILC3s and their specific role in ameliorating colitis merit exploration in future studies. Additionally, we observed a higher propionate level in the DSS+DKT group compared to the DSS group and an unchanged propionate concentration upon DSS administration compared to the healthy controls. Taken together with the upregulation of GPR43-encoding *Ffar2* mRNA among the SCFA-sensing GPCRs, it raises the possibility that GPR43 might be involved in the propionate-mediated anti-inflammatory functions through RORγt^high^-ILC3s in experimental colitis.

Unlike western medicine, herbal formulae are conventionally considered multi-target medicines as they comprise numerous chemically diverse compounds (3, 6). Besides ILC3s, our analyses suggest that DKT might be capable of directly modulating colonic epithelial cells and intraepithelial lymphocytes to protect the barrier functions. Our finding also adds more convincing evidence to previous *in vitro* experiments, which suggested that DKT enhances endogenous adrenomedullin production in intestinal epithelial cells (14). Thus, investigating the bioactive ingredients in DKT that interact with ILC3s, epithelial cells, and intraepithelial lymphocytes and identifying the DKT-activated receptors on those cells are of particular interest for future studies. In addition, experiments using germ-free and gnotobiotic animals to study the biomedical effects of DKT in experimental colitis may expand our understanding on the causative roles of gut microbiota and their metabolites.

In summary, we show that DKT blunts the severity of experimental colitis in mice by reshaping gut microbiota, enhancing propionate, and maintaining the RORγt^high^-ILC3 population. Our findings highlight novel effects of DKT on the microbiota-immune cell axis in experimental colitis. Our work provides immunological insights into DKT, serving as an anti-inflammatory agent that complements the standard western medicine-based treatment of IBD. Our study also suggests that ILC3 may serve as a potential therapeutic target for IBD. Although the biological evaluation of herbal medicine is complicated and challenging due to the lack of appropriate evaluation methods (6), our work provides a rationale and basis for future mechanistic studies of herbal medicines.

## Methods

### Mice

C57BL/6N female mice at 6-7 weeks of age were purchased from CLEA Japan, Inc. Mice were acclimatized with a normal chow diet CA-1 (CLEA Japan Inc.) for three weeks under specific-pathogen-free (SPF) conditions in the animal facility of RIKEN Yokohama Branch before the experiments. *Rorc^fl/fl^* mice were purchased from Jackson Laboratory. *Rorc^GFP/+^* mice from Dr. D. Littman (54) and CD127-Cre mice from Dr. HR. Rodewald (55) were kindly provided by the indicated investigators. To obtain specific depletion of ILC3, *Rorc^fl/fl^* mice were bred with CD127-Cre transgenic mice at the RIKEN animal facility and two parental littermates were used for the experiments. *Rorc^GFP/+^* mice were bred on a C57BL/6J background at the RIKEN SPF animal facility. For ILC3 cell sorting and cytokine detection, sex-matched littermates of *Rorc^GFP/+^* mice were used. All the transgenic mice were used at 8-12 weeks of age. The SPF facility of RIKEN is maintained in a 12-hour light, 12-hour dark cycle at 23 ± 2 °C with a humidity of 50 ± 10%, and food and water available *ad libitum*. All experimental procedures were approved by and conducted in accordance with, the Institutional Animal Care and Use Committee (IACUC) of the RIKEN Yokohama Branch.

### Dietary DKT Administration and Induction of DSS-Colitis

DKT extract granules were obtained from TSUMURA & Co. (Tokyo, Japan). We determined the final dosage of dietary DKT based on the literature (14, 56) and adjusted the human equivalent dosage to the mouse dosage based on the animal surface area (57, 58). As for the preparation of dietary DKT, we mixed the autoclaved DKT extracts and filtered maltose monohydrate powder (Wako) and blended them in a ratio of 1:8 based on previous reports (14). We then mixed them with the CA-1 powder at a defined quantity of 50g DKT powder/Kg (5% wt/wt).

For colitis experiments, mice were administered dietary DKT (DKT and DSS+DKT group) or CA-1 diet (Control and DSS group) with drinking water *ad libitum* for 6 days prior to colitis induction and continued throughout the experiments. 2.5% DSS (36,000-50,000mw; M.P. Biomedicals) was subsequently added to their drinking water and continued for five consecutive days, followed by three days of DSS-free period (**Figure 1A**). We repeated most of the experiments at least twice but mainly three times, and three to five mice were used in each experimental group. We monitored the clinical parameters during the experiments, including body weight loss, stool formation, rectal bleeding, total behavior, conditions of the fur, and survival rate until the mice were sacrificed. The severity of colitis was judged based on the previously described clinical colitis scoring system of DSS-induced colitis (59) (**Table 1**). All clinical scorings were conducted in a blinded manner. Post-mortem, the colon was dissected from cecum to anus, and the colon length was measured.

**Table 1:** Clinical colitis scoring system (59).

Clinical parameters		Score
Stool consistency	Normal, soft, soft with blood	0-2
Posture	Normal to hunched	0-2
Spontaneous behavior	Normal to no activity (without disturbing)	0-2
Provoked behavior	Normal to no activity (after disturbing)	0-2
Evaluation of the eyes	Clearness, openness	0-3
Evaluation of the fur	Cleanliness, gloss, smoothness	0-3
General appearance	Not, mildly, moderately, severely disturbed	0-3
Total highest score		17

### Histological and Immunohistochemistry

For histological analysis, 0.5 cm distal colon was fixed in Zinc Formalin (Polyscience Inc.) for 3 hours and then embedded in paraffin blocks. We then prepared 5 μm paraffin cross-sections, which were used for hematoxylin and eosin (H&E) staining (Mayer’s Hematoxylin solution, 1% Eosin Solution, Wako) following standard procedures. The histological images were captured using the BX51-P Polarizing microscope (Olympus) and processed with the Olympus D.P. Controller 2002 software.

For immunohistochemistry analysis, 0.5 cm distal colon was fixed overnight in 4% paraformaldehyde (PFA) (Wako) at 4°C and mounted in embedding medium Tissue-Tek O.C.T Compound (Sakura). The tissues were cut into 8 μm sections and permeabilized with 0.2% saponin (Nacalai Tesque) in PBS. The sections were then blocked with 5% goat serum (Wako) for Mucin 2 detection. Subsequently, the sections were stained with anti-Mucin2 (1:200, rabbit, clone: H-300, Santa Cruz Biotechnology) at 4°C overnight. For the second antibody, Alexa Fluor 488 conjugated donkey anti-rabbit IgG antibody (1:400, Thermo Fisher Scientific) was used with DAPI (1:1000, Dojindo). The sections were assessed using the Leica TCS SP8 (Leica Microsystems).

### Analysis of the Microbiota Composition in Mice Colonic Contents

The colonic contents were collected from the mice post-mortem after the colon had been removed and cut open longitudinally. Bacterial DNA was extracted as previously described with minor modifications (60). For 16S amplicon sequencing, the V4 region of 16S rRNA genes was amplified by PCR with dual barcoded primers, as previously reported (61). Sequencing of the 16s rRNA was performed on a MiSeq instrument (Illumina, 2 × 250-bp paired-end reads). Sequence data were demultiplexed using bcl2fastq v.1.8.4, then subjected to microbiome informatics using QIIME v2021-2. Taxonomy was assigned to amplicon sequence variants (ASVs) using the Silva rRNA database. For detailed analyses, ASV tables were rarefied to 20,000 reads per sample or the lowest reads within the examined dataset. Relative abundance, Bray Curtis distances, and permutational MANOVA (Adonis) were calculated with the QIIME 2 and R package qiime2R v 0.99.5, phyloseq v2, vegan v2.5-7 by RStudio v1.4.1106.

### Isolation of Colonic Epithelial Cells and Immune Cells From the Lamina Propria Region

Colons were dissected and fat tissue was removed. Colons were cut open longitudinally and washed with cold RPMI-1640 medium (Sigma Aldrich) to remove luminal contents and debris, then incubated in RPMI-1640 medium containing 5mM EDTA and 2% fetal bovine serum (FBS) for 15 mins at 37°C, followed by 2% FBS in RPMI-1640 medium for another cycle. After vigorously shaking for 15s, the dissociated cells were collected as colonic epithelial cells. The epithelial cells were then passed through a 40 μm cell strainer (B.D. Biosciences) and cell pellets were stored after being washed with cold PBS. For the immune cell isolation from the lamina propria region, the remaining colonic tissues were cut into small pieces and digested with 1.0 mg/ml collagenase (Sigma) suspended in RPMI-1640 medium for 15 min at 37°C. The resultant supernatants from the collagenase digestion were collected and passed through a 100 μm cell strainer after 3 cycles of these steps. The cells were subjected to Percoll (G.E. Healthcare) gradient separation and lymphocytes in the interphase were collected and proceeded for the flow cytometric analysis.

### Flow Cytometric Analysis and Cell Sorting

Single-cell suspensions (1×10^6^ cells/sample) were stained with the indicated antibodies at 4°C after blocking Fc receptors with the 2.4G2 mAb (BD Pharmingen) and dead cells were stained with LIVE/DEAD fixable Aqua Dead Cell Stain (Thermo Fisher). For detection of ILC3, CD4^+^ T cells, B cells, DCs and macrophages, we used fluorochrome-conjugated antibodies, all from BioLegend, against combinations, indicated below, of the following surface antigens: CD45.2 (PerCP/Cyanine5.5, clone:104), CD3ϵ (FITC or BV605, clone: 145-2C11), TCR β (FITC or BV605, clone: H57-597), CD4 (APC/Cyanine7, clone: GK1.5), CD19 (BV605, clone: 6D5), CD127 (PE/Cyanine7, clone: A7R34), NKp46 (FITC, clone: 29A1.4), MHC class II (FITC or BV421, M5/114.15.2), F4/80 (FITC, clone: BM8), CD103 (PE/Cyanine7, clone: 2E7), CD11b (APC, M1/70), Ly6C (APC/Cyanine7, clone: HK1.4). We also used CCR6 (PE, Clone: 140706, R&D Systems) and CD11c (PE, clone: HL3, BD). CD4^+^ T cells were identified as CD45.2^+^CD3ϵ^+^TCRβ^+^CD4^+^CD19^-^; B cells were gated as CD45.2^+^CD3^-^TCRβ^-^CD19^+^; conventional DCs were gated as CD45.2^+^CD11bϵ^+^CD11c^high^MHCII^+^; macrophages were gated as CD45.2^+^CD11c^-^CD11b^+^F4/80^inter^Ly6C^low^CD64^+^MHCII^+^.

For transcription factor staining, after staining with antibodies to surface antigens as described above, the lymphocytes were then fixed and permeabilized with Foxp3/Transcription Factor Buffer set (Thermo Fisher Scientific) according to the manufacturer’s instructions and stained with T-bet (APC, clone: 4B10, BioLegend), GATA3 (PE, clone: 16E10A23, BioLegend) and RORγt (BV421, clone: Q31-378, BD). ILC1/NK cells were gated as CD45.2^+^CD3ϵ^-^TCRβ^-^CD19^-^CD127^+^GATA3^-^RORγt^-^T-bet^+^; ILC2 were gated as CD45.2^+^CD3ϵ^-^TCRβ^-^CD19^-^CD127^+^GATA3^high^RORγt^-^: ILC3 were gated as CD45.2^+^CD3ϵ^-^TCRβ^-^CD19^-^CD127^+^GATA3^low^RORγt^+^; Th17 were gated as CD45.2^+^CD3ϵ^+^TCRβ^+^ CD19^-^CD4^+^RORγt^+^.

For the measurement of intracellular cytokine production, cells were cultured for 3 hr at 37°C in the presence of GolgiPlug (BD Biosciences). The cells were subsequentially stained with IL-22 (PE, clone: Poly5164, BioLegend) and IL-17A (APC, clone: TC11-18H10.1, BioLegend) antibodies after being fixed with 4% PFA.

For colonic ILC3 sorting, *Rorc^GFP/+^* mice were used. CD45.2^+^CD3ϵ^-^TCRβ^-^CD19^-^CD127^+^ *Rorc^GFP^* ^-high^ expressing ILC3s were gated and sorted to detect cytokines and GPCR mRNAs by qPCR.

All stained cells were analyzed or sorted on a BD FACSAriaIII. For all lymphocyte analysis by flow cytometry, lymphocytes were first strictly defined by forward scatter (FSC), and side scatter (SSC) intensity and then carefully gated based on CD45 expression. The data were analyzed with FlowJo v10.8.0.

### RNA Extraction and Quantitative PCR Analysis of Colonic Epithelial Cells and ILC3

The epithelial cells were lysed in RLT buffer (QIAGEN) with 2-mercaptoethanol (Nacalai Tesque) after washing with PBS. The RNeasy Mini Kit (QIAGEN) was used for total RNA extraction following the manufacturer’s instructions. cDNA was synthesized through reverse transcription PCR with SuperScript IV Reverse Transcriptase (Thermo Fisher Scientific) using random hexamer primer (Thermo Fisher Scientific).

For the colonic ILC3 sorting, 200-500 colonic ILC3 isolated from *Rorc^GFP/+^* mice were directly sorted into RLT buffer with 2-mercaptoethanol (Nacalai Tesque). Total RNA from sorted ILC3 was extracted with RNeasy Micro Kit (QIAGEN) following the manufacturer’s instructions. The extracted RNA was subsequentially subjected to amplification with a SuperScript™ IV Single Cell/Low Input cDNA PreAmp Kit (Thermo Fisher Scientific).

Real-time PCR for epithelial cells and ILC3s was performed with SYBR Premix Ex Taq (Takara) on a LightCycler 480 (Roche). The primers were used for the analyses are shown in **Table 2**.

**Table 2:** Primers used in the study.

Primer	sequence
*Hprt*	Forward, 5’-TCAGTCAACGGGGGACATAAA-3’
	Reverse, 5’-GGGGCTGTACTGCTTAACCAG-3’
*Muc2*	Forward, 5’-TCAGCACACCAACCAAAACC-3’
	Reverse, 5’-CACTTCAGCGGCACAATCTC-3’
*Reg3g*	Forward, 5’-ACCTAGCCACAAGCAAGATCCCA-3’
	Reverse, 5’- ATGAGAGGAGGGAAGGGCCA-3’
*Fut2*	Forward, 5’-GAGTCAAGGGGAGGGAGAAC-3’
	Reverse, 5’-AACTTGGTGAGGGGACTGTG-3’
*Il-22*	Forward, 5’-CATGCAGGAGGTGGTACCTT-3’
	Reverse, 5’-CAGACGCAAGCATTTCTCAG-3’
*Il17a*	Forward, 5’-TCCAGAAGGCCCTCAGACTA-3’
	Reverse, 5’-TGAGCTTCCCAGATCACAGA-3’
*Ffar2(Gpr43)*	Forward, 5’-CTTGATCCTCACGGCCTACAT-3’
	Reverse, 5’-CCAGGGTCAGATTAAGCAGGAG-3’
*Ffar3 (Gpr41)*	Forward, 5’ -ACCTGACCATTTCGGACCT-3’
	Reverse, 5’-CCATCTCATGCCACATGC-3’
*Hcar2 (Gpr109a)*	Forward, 5’ -ATGGCGAGGCATATCTGTGTAGCA-3’
	Reverse, 5’-TCCTGCCTGAGCAGAACAAGATGA-3’

### Quantification of Fecal SCFAs

SCFAs and other metabolites were extracted and measured as previously described (60). In brief, 5 mg of feces or colonic contents were lyophilized. Dried samples were added to 5 μl Milli-Q water containing internal standards (2.2 mM [1,2-13C2]acetate, 2.2 mM [2H7]butyrate and 2.2 mM crotonate), 50 μl HCl and 200 μl diethyl ether. After centrifugation, 80 μl of the organic layer was transferred to a glass vial and then 16 μl of N-tert-butyldimethylsilyl-N-trifluoroacetamide (Sigma-Aldrich) was added to derivatize the samples. The vials were incubated at 80°C or 20 min and allowed to stand for 48h before injection. The analysis was performed using a gas chromatography-tandem mass spectrometry (GCMS) platform on a Shimadzu GCMS-TQ8040 triple quadrupole mass spectrometer (Shimadzu) with a capillary column (BPX5) (SGE Analytical Science). The program of GCMS analysis is described in a previously published paper (60). The GCMS data were processed, and concentrations were calculated by LabSolutions Insight (Shimadzu).

### Statistical Analysis

Multiple sample comparisons were performed by one-way ANOVA with Tukey’s *post hoc* test. All statistical analyses were performed using Prism version 8 and 9 (GraphPad Software, USA). P < 0.05 and FDR adjusted P < 0.05 were considered statistically significant.

### Diagram

Created with BioRender.com, as shown in **Supplementary Figure 3**.

## Data Availability Statement

The data presented in the study are deposited in the DDBJ repository, accession number: DRA013869, run: DRR359766-DDR359820. The data can be found at the link: https://ddbj.nig.ac.jp/resource/sra-submission/DRA013869.

## Ethics Statement

The animal study was reviewed and approved by the Institutional Animal Care and Use Committee (IACUC) of the RIKEN Yokohama Branch.

## Author Contributions

ZS, NS-T and HO conceived the study. ZS designed, performed the experiments and data analyses and co-wrote the manuscript. TT contributed to the microbiota data analyses and data interpretation. YN quantified the SCFA concentrations in the mouse colons using GCMS. TKato performed 16S rRNA gene sequencing for the mouse colonic contents. KB and RN contributed to the experiments and helped to interpret the data. TKageyama contributed to the flow cytometry analyses and cell sorting. AI assisted with the SCFA analyses. NS-T and HO directed the research, provided essential materials and co-wrote the manuscript. All authors contributed to the article and approved the submitted version.

## Funding

ZS, TT and RN are recipients of RIKEN Junior Research Associate (JRA) Program. KB was supported by Deutsche Forschungsgemeinschaft (DFG; BE 6909/1-1). NS-T was supported by the Japan Society for the Promotion of Science KAKENHI (20KK0360), the Japan Agency for Medical Research and Development AMED-PRIME and Yakult Bio-Science foundation. HO was supported by the Japan Society for the Promotion of Science KAKENHI (19H01030, 19F19785).

## Conflict of Interest

The authors declare that the research was conducted in the absence of any commercial or financial relationships that could be construed as a potential conflict of interest.

## Publisher’s Note

All claims expressed in this article are solely those of the authors and do not necessarily represent those of their affiliated organizations, or those of the publisher, the editors and the reviewers. Any product that may be evaluated in this article, or claim that may be made by its manufacturer, is not guaranteed or endorsed by the publisher.

## Supplementary Material

Supplementary Figure 1 The relative abundance of several family-level bacteria in the four experimental groups. The relative abundance of fecal Akkermanciaceae, Clostridiaceae, Bacteriodaceae, and Eggerthellaceae (Control N=15, DKT N=15, DSS N=14, DSS+DKT N=11). Each symbol represents data from an individual mouse. Data are pooled from 3 independent experiments with three to five mice in each experimental group. Graphs show means ± SEM; *p<0.05, **p<0.01, and ***p<0.001. Statistical analysis was performed using One-way ANOVA with Tukey’s multiple comparisons test.

Supplementary Figure 2 DKT treatment does not affect ILC1, ILC2, T and B lymphocytes, macrophages, or DCs. **(A)** Gating strategy for colonic ILC3. For flow cytometry analyses, lymphocytes were firstly gated on SSC-A vs. FSC-A and doublets were excluded by gating on SSC-W vs. SSC-H. Live immune cells were further gated as Zombie dye negative and CD45 positive cells. CD127 expression was used to gate the ILC population. **(B)** The absolute cell number of colonic ILC, ILC1/NK cells and ILC2 (Control N=7, DKT N=8, DSS N=8, DKT+DSS N=8). **(C)** The absolute cell number of colonic CD4^+^ T cells, B cells, macrophages, and DCs (Control N=7, DKT N=8, DSS N=8, DKT+DSS N=8). **(D)** Relative expression of *Il22* and *Il17a* in RORγt^high^-ILC3s using *Rorc^GFP/+^* mice (Control N=4, DKT N=4, DSS N=5, DSS+DKT N=4). **(E)** Relative expression of G-protein coupled receptors (GPCRs), *Ffar2, Ffar3* and *Hcar2* mRNAs in RORγt^high^-ILC3s using *Rorc^GFP/+^* mice (Control N=4, DKT N=5, DSS N=5, DSS+DKT N=5). Each symbol **(B–E)** represents data from an individual mouse. Graphs **(A–C)**, Results are pooled from two independent experiments with three to four mice in each experimental group. Graph **(E)**, results are representative of three independent experiments. Graphs **(B–E)** display means ± SEM; *p<0.05, **p<0.01, and ***p<0.001. Statistical analysis was performed using One-way ANOVA with Tukey’s multiple comparisons test.

Supplementary Figure 3 Graphical abstract summarizing the observations described in this paper. DKT treatment sustains colonic homeostasis by changing the activities of luminal microbes, enhancing RORγt^high^-ILC3s and protecting barrier function.
